# The Usefulness of Homeostatic Measurement Assessment-Insulin Resistance (HOMA-IR) for Detection of Glucose Intolerance in Thai Women of Reproductive Age with Polycystic Ovary Syndrome

**DOI:** 10.1155/2012/571035

**Published:** 2012-04-24

**Authors:** Thanyarat Wongwananuruk, Manee Rattanachaiyanont, Pichai Leerasiri, Suchada Indhavivadhana, Kitirat Techatraisak, Surasak Angsuwathana, Prasong Tanmahasamut, Chongdee Dangrat

**Affiliations:** Gynecologic Endocrinology Unit, Department of Obstetrics and Gynecology, Faculty of Medicine Siriraj Hospital, Mahidol University, Bangkok 10700, Thailand

## Abstract

*Objectives*. To study the cut-off point of Homeostatic Measurement Assessment-Insulin Resistance (HOMA-IR) as a screening test for detection of glucose intolerance in Thai women with polycystic ovary syndrome (PCOS). *Study Design*. Cross-sectional study. *Setting*. Department of Obstetrics and Gynecology, Faculty of Medicine Siriraj Hospital. *Subject*. Two hundred and fifty Thai PCOS women who attended the Gynecologic Endocrinology Unit, during May 2007 to January 2009. *Materials and Methods*. The paitents were interviewed and examined for weight, height, waist circumference, and blood pressure. Venous blood samples were drawn twice, one at 12-hour fasting and the other at 2 hours after glucose loading. *Results*. The prevalence of glucose intolerance in Thai PCOS women was 20.0%. The mean of HOMA-IR was 3.53  ±  7.7. Area under an ROC curve for HOMA-IR for detecting glucose intolerance was 0.82. Using the cut-off value of HOMA-IR >2.0, there was sensitivity at 84.0%, specificity at 61.0%, positive predictive value at 35.0%, negative predictive value at 93.8%, and accuracy at 65.6%. *Conclusion*. HOMA-IR >2.0 was used for screening test for glucose intolerance in Thai PCOS women. If the result was positive, a specific test should be done to prove the diagnosis.

## 1. Introduction

Polycystic ovary syndrome (PCOS) is one of the most common endocrine disorders, affecting around 4–7% of the population in women of the reproductive age [1]. Diagnostic criteria for PCOS mostly use the revised Rotterdam 2003 criteria [2]. However, the etiology and pathophysiology of PCOS remain unclear, and the multiple risk factors such as genetics, environment, nutrition, lifestyle, and much more are still under investigation. There is heterogeneity of symptoms and in severity of disease but most have central obesity or android fat deposition (fat at abdominal wall and viscera). Android fat deposition is relatively resistant to insulin hormone [3, 4]. According to International Diabetes Federation 2005 criteria for metabolic syndrome (IDF 2005) [5], central obesity is diagnosed when waist circumference is more than 80 centimeters for Asian women. Both of central obesity and hyperandrogenism in PCOS aggravate insulin resistance which promotes incidence of diabetes mellitus. Several studies showed that overall abnormal oral glucose tolerance test (OGTT) in PCOS is 42–45% which impair glucose tolerance test 25–31%, and diabetes mellitus 7.5–10%, and all the abnormalities are associated with age, higher body mass index (BMI), central obesity, and hyperandrogenemia [6–9]. Acanthosis nigricans is a brown to black, poorly defined, velvety hyperpigmentation of the skin, usually present in the posterior and lateral folds of neck, axilla, groin, and other areas. The most common cause would be insulin resistance [10]. By a precisely unknown pathophysiology, it associates with obesity, and found in PCOS women is about 7.5–74% [11–13]. Detection of glucose intolerance in PCOS is achieved by many methods. Hyperinsulinaemic euglycaemic clamp technique is the gold standard of insulin sensitivity measurements. However, this technique is very complex and requires experienced personnel, two intravenous lines throughout the study, and frequent bedside plasma glucose determinations, so this technique is not appropriate in clinical practice [14]. Rotterdam ESHRE/ASRM-sponsor PCOS Consensus Workshop Group suggests the oral glucose tolerance test (OGTT) has been traditionally used for the diagnosis of diatbetes [14], by using 75 gram glucose load and measure glucose at fasting and 2 hours postloading of glucose. These diagnostic criteria are followed by American Diabetes Association (ADA) 2007 [15].

 Practically, we have been trying to find a solution to measure insulin resistance by considering the concept that patients who have insulin resistance will have more insulin hormone in blood than those who does not. Since insulin cannot perform its duty well, this causes for higher level of glucose in blood. Homeostatic Measurement Assessment Insulin Resistance (HOMA-IR) is an evaluation of insulin resistance by using glucose level in blood and insulin of patients while fasting to analysis. This evaluation can be also used to find the insulin resistance which has more advantage than the 75 gram OGTT that a person who receives the test is drawn blood only one. It might has benefit for screening. From the previous studies, there is still no conclusion about cut-off point used in diagnosis of insulin resistance. Matthews et al. used HOMA-IR more than 2.5 for diagnosis of insulin resistance in general population [16]. Keskin et al. studied in pubertal obese children and adolescents; this study has shown HOMA cut-off point at 3.16 [17]. Jensterle et al. used cut-off point for HOMA-IR of at least 2.0 for diagnosis of insulin resistance in young PCOS women [18]. Most studies of HOMA-IR in PCOS women were in American and European women. HOMA-IR did not have the precise cut-off value used in diagnosis of insulin resistance. Therefore, the objective of this study was to determine the result of using HOMA-IR as a diagnostic test for detection of abnormal OGTT in Thai women with PCOS, for discovering the proper cut-off value to diagnose glucose intolerance and getting the data important for applying in clinical practice.

## 2. Materials and Methods

This cross-sectional study was conducted. Using data is based on the records of 250 PCOS women who consecutively attended the Gynecologic Endocrinology Unit of the Department of Obstetrics and Gynecology, Siriraj Hospital between May 2007 and January 2009, which were reviewed and analyzed. The diagnosis of PCOS was defined by the Revised Rotterdam Criteria 2003 [2].

Exclusion criteria included the women who had previous surgery of one or both ovaries, used hormonal treatment, and took the medication for dyslipidemia within 3 months and/or received steroid within 6 months before participation in the study. The study protocol was approved by the Ethics Committee of the Faculty of Medicine Siriraj Hospital, Mahidol University. This study was financially supported by Siriraj Routine to Research (R2R) Management Fund.

All the women with PCOS who participated in this study received a physical examination including measurement of vital signs and skin lesions, and anthropometric measurements, as a prelude to a review of clinical presentations. Age, body weight, height, waist circumference, blood pressure, and skin manifestations were recorded. After overnight fasting for at least 12 hours, venous blood samples were drawn twice, the first one at 8–10 AM and the second one at 2-hour postglucose loading to measure glucose and insulin level at baseline and 2 hours following oral 75 g glucose loading. The first blood sample was also examined for baseline hormonal profiles (prolactin, cortisol, thyroid stimulating hormone (TSH), and androgen hormone) and baseline metabolic profile (glucose, insulin, and lipid). The second blood sample was examined for glucose and insulin postglucose loading. 

### 2.1. Diagnostic Criteria

PCOS was diagnosed by the Revised Rotterdam Criteria 2003 [2], that is, including a patient who has at least 2 in 3 of the following: (1) oligomenorrhea or amenorrhea, (2) hyperandrogenemia and/or hyperandrogenism, (3) polycystic ovaries, and excluding another causes (e.g., hyper/hypothyroidism, hyperprolactinemia, Cushing's syndrome, congenital adrenal hyperplasia (CAH), or hormonal secreting tumor).

 Glucose tolerance was evaluated using 75 g OGTT according to the American Diabetes Association (ADA) 2007 criteria [15]. Abnormal OGTT is classified as follows: (i) impaired fasting glucose (IFG), that is, fasting glucose (FG) ≥100 and <126 mg/dL, (ii) impaired glucose tolerance test (IGT), that is, 2 hr glucose ≥140 and <200 mg/dL, (iii) type 2 Diabetes mellitus (DM), that is, fasting blood glucose ≥126 mg/dL and/or 2 hr glucose ≥200 mg/dL. In our study, glucose intolerance was composed of IFG, IGT, and Type 2 DM.

The HOMA-IR was calculated by multiplying fasting Insulin (U/mL) by fasting glucose (mmol/L) and dividing by 22.5.

Obesity was defined as body mass index (BMI) ≥25 kg/m^2^ according to the WHO cut-off points for Asian populations [19]. Central obesity was defined as WC ≥80 cm according to the International Diabetes Federation (IDF) 2005 [5].

Hyperandrogenemia was defined as total testosterone >0.8 ng/mL, or free testosterone >0.006 ng/mL, or dehydroepiandrosterone sulphate (DHEAS) >350 microgram/dL [20].

### 2.2. Laboratory Assays

All laboratory assays were performed at the laboratory unit of Department of Clinical Pathology, Faculty of Medicine Siriraj Hospital, Mahidol University, the central laboratory certified by IS0 15189. All assays were done using an automatic analyzer (Modular P800, Roche; for glucose and Modular E170, Roche; for insulin, TSH, prolactin, cortisol). All techniques had intra- and interassay coefficients of variation (CV) less than 5%.

### 2.3. Statistical Analysis

 Statistical analysis was performed using SPSS version 13 (SPSS Inc.). Data were presented in mean ± SD or number (%) as appropriate. The sensitivity specificity positive predictive value, negative predictive value, and diagnostic accuracy were calculated from 2 × 2 tables for HOMA-IR at each cut-point for detection of abnormal OGTT. Receiver operator curves (ROCs) for HOMA-IR and abnormal OGTT were created by calculating the sensitivity and specificity of fixed cut-off points of the various parameters examined.

## 3. Results

 A total of 250 women with PCOS were studied. The clinical and laboratory characteristics were summarized in Table 1. The mean age, BMI, and waist circumference (WC) were 25.4 ± 5.8 years old, 26.2 ± 7.6 kg/m^2^ and 82.3 ± 16.3 cm, respectively. The diagnostic criteria of PCOS were 98.4% of oligomenorrhea, 49.2% of hyperandrogenism, and 97.2% of polycystic ovaries. In this study 27.2% had acanthosis nigricans. The blood level of carbohydrate metabolic profiles showed the fasting blood glucose was 85.4 ± 22.9 mg/dL, 2 hr blood glucose was 116.4 ± 53.8 mg/dL, fasting insulin was 15.6 ± 34.2 mu/mL, 2 hr insulin was 106.6 ± 89.0 mu/mL, and HOMA-IR was 3.53 ± 7.74.

 The prevalence of an glucose intolerance is shown in Table 2. An glucose intolerance was found in 20.0%, with 5.6% having type 2 diabetes mellitus, 3.2% having impaired fasting glucose levels, and 13.6% having an impaired glucose tolerance test.

**Figure 1:**
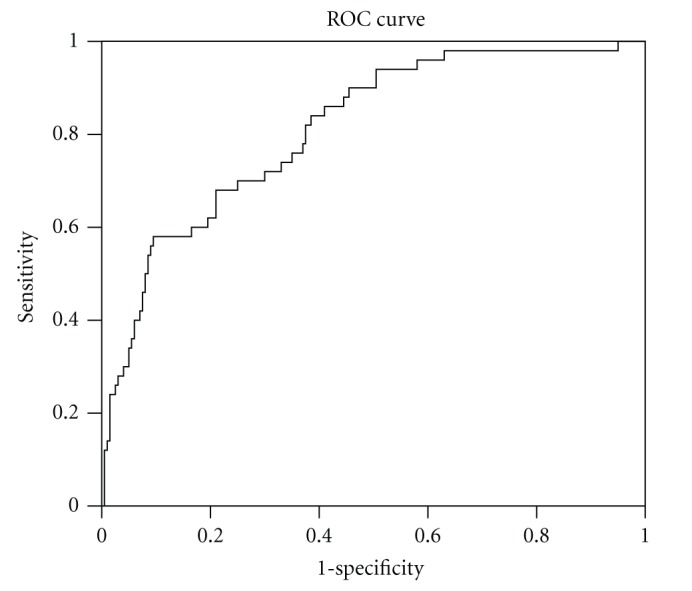
ROC curve for HOMA-IR for detecting glucose intolerance in Thai PCOS women. The area under the curve is 0.82.

Figure 1 showed ROC curve of HOMA-IR and glucose intolerance. The area under the curve was 0.82.

Sensitivity, specificity, positive predictive value, negative predictive value, and accuracy of the cut-off HOMA-IR of more than 2.0 for detection of glucose intolerance in PCOS women were 89.74%, 58.82%, 38.46%, 95.24%, and 65.71%, respectively, as in Table 3.

Table 4 shows the relation between associating factors of glucose intolerance in PCOS and HOMA-IR >2.0. All had statistical significance with HOMA-IR >2.0. Odds ratio for age ≥30 = 2.45, BMI ≥25 = 23.37, WC ≥80 cm = 24.55, hyperandrogenism = 1.97, and presence of acanthosis nigricans = 50.03. 

Using cut-off point HOMA >2.0 and the number of clinical associating factor for insulin resistance, odds ratio in each condition is shown in Table 5. The significant odds ratio included the conditions of HOMA >2.0 combined with 4 clinical associating factors and HOMA >2.0 combined with 5 clinical associating factors; odds ratio (95% CI) were 2.67 (1.31–5.42) and 9.75 (3.16–30.10), respectively.

## 4. Discussions

 Insulin resistance and the consequent development of hyperinsulinemia seem to be an important pathophysiological mechanism that links PCOS to its concurrent metabolic derangements [1]. An insulin resistance is due to alterations in *β*-cell function, it might have a key role in the impaired glucose tolerance test and the development of frank diabetes in women with PCOS. It is well known that type 2 DM is an important risk factor for coronary heart disease. In previous studies, type 2 DM was found to contribute significantly to the mortality of women with PCOS (odds ratio 3.6; 95% confidence interval 1.5–8.4) more than that expected in unaffected women [21]. In this study, our data indicated that the prevalence of abnormal glucose tolerance in our study was lower than that of American women with PCOS but similar to that of Chinese women with PCOS [22]. According to some, differences among these studies in the selection criteria of PCOS cannot be ignored and the factors of ethnic background, dietary composition, and lifestyle might play an important role in the prevalence of abnormal glucose tolerance in women with PCOS.

 Therefore, an OGTT is currently the only reliable way to detect impaired glucose metabolism in PCOS. This procedure is relatively time-consuming and inconvenient for the patient, which limits its use as a general screening instrument in daily practice. Therefore, a more convenient screening test that minimizes the need for an OGTT is desirable. In this study, HOMA-IR had the close relation for detection of abnormal OGTT in Thai PCOS women. If using the cut-off level of HOMA-IR >2.0 for detecting the glucose intolerance, it can give more sensitivity, but specificity was less than the higher cut-off level. It may be suitable for use as a screening test for detecting of glucose intolerance or insulin resistance in Thai PCOS women. In the cases which had a false positive test, if they received the treatment to control insulin intolerance, it did not have a serious effect on the treatment. Because the early step of treatment is life style modification, control of body weight, control of diet and exercise, these methods are the better way for controlling other metabolic disorders, too. Insulin sensitizing drug for example, metformin, provides benefit to control insulin resistance in PCOS women. Nevertheless this drug would give minor gastrointestinal side effect and no serious adverse event was reported [23].

Many studies use the HOMA-IR as the diagnostic criteria for insulin resistance [2, 17, 18, 24]. The European Group for the Study of Insulin Resistance (EGIR) uses the cut-off level of HOMA-IR >2.0 to indicate insulin resistance or glucose intolerance [24]. Some studies used different cut-off levels of HOMA-IR, because of the differences of each ethnic group, the prevalence of obesity or central obesity or age group. And no consensus on the cut-off level of HOMA-IR exists for Thai PCOS women. A screening strategy that uses BMI and waist circumference, which is a low-cost and rapidly performed approach, could save about 23% of OGTT [25]. However, if PCOS women have the risk factors to develop insulin resistance, the diagnostic procedure should be done.

 Many studies showed clinical risk factors of glucose intolerance in PCOS: age, BMI, central obesity, hyperandrogenism, presence of acanthosis nigricans [6–9, 26–28]. Table 4 shows a statistical relation between HOMA-IR >2.0 and these factors. If PCOS women presented clinical risk factor and had abnormal HOMA-IR, especially 4 and 5 clinical risks, these women would have a significant odds ratio to have glucose intolerance (Table 5). On the other hand, we can use the clinical risk to select high risk women for investigation of a specific test for glucose intolerance (75 g OGTT).

Limitations of this study were that it was cross-sectional study and had no control group. The majority of population in this study was younger and was an urban population; this might not be able to fully represent all Thai PCOS women. To overcome these limitations, a prospective, multicenter study is needed.

## 5. Conclusion

 HOMA-IR was an easily obtainable, safe, low cost, and less invasive test than OGTT. HOMA-IR >2.0 was used as a screening test for glucose intolerance in Thai PCOS women. If the result was positive and had many clinical risk factors, a specific test should be done to prove the diagnosis.

## Figures and Tables

**Table 1:** Characteristics of 250 PCOS Thai women.

Characteristics	Mean ± SD or *n* (%, 95% CI)
Age (yr)	25.4 ± 5.8
Body mass index (kg/m^2^)	26.2 ± 7.6
Waist circumference (cm)	82.3 ± 16.3
Systolic blood pressure (mmHg)	112.5 ± 12.5
Diastolic blood pressure (mmHg)	70.3 ± 9.1
Presence of acanthosis nigricans	68 (27.2, 21.7–32.7)
Carbohydrate metabolism	
Fasting plasma glucose (mg/dL)	85.4 ± 22.9
Fasting plasma insulin (mu/mL)	15.6 ± 34.2
2 hour plasma glucose (mg/dL)	116.4 ± 53.8
2 hour plasma insulin (mu/mL)	106.6 ± 89.0
HOMA-IR	3.53 ± 7.74
Lipid profiles	
Cholesterol (mg/dL)	189.2 ± 37.6
Triglyceride (mg/dL)	103.2 ± 66.2
High density lipoprotein cholesterol (mg/dL)	55.4 ± 14.6
Low density lipoprotein cholesterol (mg/dL)	112.0 ± 32.5
Androgen profiles	
Total testosterone (ng/mL)	0.735 ± 0.388
Free testosterone (ng/mL)	0.014 ± 0.009
DHEAS (microgram/dL)	256.8 ± 107.2

HOMA-IR: Homeostatic Measurement Assessment-Insulin Resistance, DHEAS: dehydroepiandrosterone sulphate.

**Table 2:** Prevalence of glucose intolerance in 250 Thai women with polycystic ovary syndrome.

Glucose intolerance^†^	Prevalence	
	*n*	% (95% CI)
Overall	50	20.0 (15.04–24.96)
Impaired fasting glucose (IFG)^‡^	8	3.2 (1.02–5.38)
Impaired glucose tolerance (IGT)	34	13.6 (9.35–17.85)
Diabetes mellitus (DM)	14	5.6 (2.75–8.45)

^†^Glucose intolerance: impaired fasting glucose (fasting plasma glucose ≥100 and <126 mg/dL), impaired glucose tolerance test (2 hr glucose ≥140 and <200 mg/dL) or the presence of diabetes mellitus (fasting plasma glucose ≥126 mg/dL and/or 2 hr glucose ≥200 mg/dL).

^‡^4 women had combined IFG and IGT and 2 women had combined IFG and 2 hr glucose ≥200 mg/dL.

**Table 3:** Sensitivity, specificity, positive predictive value, negative predictive value and accuracy of HOMA-IR >2.0 for detection of glucose intolerance in Thai PCOS women.

Parameter	Percent (%)	95% CI
Sensitivity	84.0	79.5–88.5
Specificity	61.0	55.0–67.1
Positive predictive Value	35.0	29.1–40.9
Negative Predictive Value	93.8	91.1–96.9
Accuracy	65.6	60.1–71.9

**Table 4:** Relation of HOMA-IR and associating factors of glucose intolerance.

Variables	HOMA-IR ≤2	HOMA-IR >2	Odds ratio	95% CI
	*n* (%)	*n* (%)		odds ratio
Age (years old)			2.45	1.33–4.53
<30	110 (57.0)	83 (43.0)		
≥30	20 (35.1)	37 (64.9)		
BMI (kg/m^2^)			23.37	12.07–45.26
<25	111 (82.2)	24 (17.8)		
≥25	19 (16.5)	96 (83.5)		
Waist circumference (cm)			24.55	12.64–47.67
<80	108 (84.4)	20 (15.6)		
≥80	22 (18.0)	100 (82.0)		
Hyperandrogenism			1.97	1.19–3.26
No	76 (60.3)	50 (39.7)		
Yes	54 (49.3)	70 (56.5)		
Acanthosis nigricans			50.03	15.07–166.08
Absent	127 (69.8)	55 (30.2)		
Present	3 (4.4)	65 (95.6)		

**Table 5:** Odds ratio in each condition which showed HOMA >2.0 with number of clinical associating factors.

Condition	*n* (%)	OR (95% CI)	*P* value
HOMA >2.0 with 1 clinical associating factor	2 (0.8)	0.79 (0.74–0.85)	0.219
HOMA >2.0 with 2 clinical associating factors	6 (2.4)	1.10 (0.42–2.89)	0.805
HOMA >2.0 with 3 clinical associating factors	11 (4.4)	1.97 (0.89–4.35)	0.113
HOMA >2.0 with 4 clinical associating factors	16 (6.4)	2.67 (1.31–5.42)	0.008
HOMA >2.0 with 5 clinical associating factors	20 (8.0)	9.75 (3.16–30.10)	<0.001

Clinical associating factors are age ≥30 year old, BMI ≥25 kg/m^2^, waist circumference ≥80 centimeters, presence of acanthosis nigricans, and hyperandrogenism.
